# Motivations for pre-exposure prophylaxis uptake and decline in an HIV-hyperendemic setting: findings from a qualitative implementation study in Lesotho

**DOI:** 10.1186/s12981-023-00535-x

**Published:** 2023-07-06

**Authors:** Joy J. Chebet, Shannon A. McMahon, Tapiwa Tarumbiswa, Hlalele Hlalele, Chivimbiso Maponga, Esther Mandara, Kacey Ernst, Halimatou Alaofe, Till Baernighausen, John E. Ehiri, Pascal Geldsetzer, Mark Nichter

**Affiliations:** 1 Department of Health Promotion Sciences, Mel and Enid Zuckerman College of Public Health, University of Arizona, Tucson, AZ USA; 2 Heidelberg Institute of Global Health (HIGH), Heidelberg University, Heidelberg, Germany; 3 Social and Behavioral Interventions, Johns Hopkins Bloomberg School of Public Health, Baltimore, MD USA; 4 Ministry of Health, Maseru, Lesotho; 5 Clinton Health Access Initiative, Maseru, Lesotho; 6 Department of Epidemiology and Biostatistics, Mel and Enid Zuckerman College of Public Health, University of Arizona, Tucson, AZ USA; 7 Department of Global Health and Population, Harvard T.H. Chan School of Public Health, Boston, MA USA; 8 Africa Health Research Institute (AHRI), Durban, South Africa; 9 Division of Primary Care and Population Health, Department of Medicine, Stanford University, Stanford, CA USA; 10 Chan Zuckerberg Biohub, San Francisco, CA USA; 11 School of Anthropology, University of Arizona, Tucson, AZ USA

**Keywords:** Pre-Exposure Prophylaxis (PrEP), Uptake, Initiation, Decline, Lesotho, Sub-Saharan Africa

## Abstract

**Background:**

Pre-Exposure Prophylaxis (PrEP) has demonstrated clinical efficacy in preventing HIV infection, yet its uptake remains low. This study, conducted in five PrEP implementing districts in Lesotho, examined factors motivating persons at risk of HIV infection to adopt or reject PrEP when offered freely.

**Methods:**

In-depth interviews were undertaken with stakeholders directly engaged with PrEP policy (n = 5), program implementation (n = 4), and use (current PrEP users = 55, former PrEP users = 36, and PrEP decliners (n = 6)). Focus group discussions (n = 11, 105 total participants) were conducted with health staff directly providing HIV and PrEP services.

**Results:**

Demand for PrEP was reported highest among those at greatest risk for HIV acquisition: those in serodiscordant relationships and/or engaged in sex work. Culturally sensitive PrEP counseling was described as an opportunity to transfer knowledge, build trust, and address user concerns. Conversely, top-down counseling resulted in PrEP distrust and confusion about HIV status. Key motivations for PrEP uptake revolved around sustaining core social relationships, desire for safer conception, and caring for ailing relatives. The decline of PrEP initiation was driven by a combination of individual-level factors (risk perception, perceived side effects, disbelief of the drug’s efficacy and PrEP’s daily pill regimen), societal factors (lack of social support and HIV-related stigma), and structural factors related to PrEP access.

**Conclusions:**

Our findings suggest strategies for effective national PrEP rollout and implementation include: (1) demand creation campaigns which highlight positive aspects of PrEP, while simultaneously addressing apprehensions for uptake; (2) strengthening health provider counseling capacity; and (3) addressing societal and structural HIV-related stigma.

## Background

Despite decades of investment in HIV/AIDS programs, the African continent continues to bear a disproportionate burden of the disease. Of the 1.5 million new cases registered globally in 2021, approximately 860,000 (57%) were recorded in the sub-Saharan African (SSA) region [1]. The course of the HIV pandemic in SSA is more uncertain than ever due to a plateauing of international donor funding to address the disease, coupled with uncertainty about the degree to which local governments will take over financing of these programs [2]. Additionally, disruption of services during the COVID-19 pandemic places the HIV/AIDS epidemic in SSA at greater peril [3].

To address the global HIV epidemic, comprehensive approaches that provide treatment to those living with HIV while simultaneously preventing new infections have been promoted [1]. The use of antiretroviral (ARV) drugs as Pre-Exposure Prophylaxis (PrEP) has demonstrated clinical efficacy in preventing HIV infection in seronegative individuals [4, 5]. Following safety and efficacy trials, once-daily oral PrEP, formulated as either tenofovir (TDF) or combination tenofovir/emtricitabine (TDF/FTC), was endorsed by the World Health Organization (WHO) in 2015 for use among HIV negative individuals at substantial risk of HIV infection [6]. If used widely in HIV-negative populations, the drug can potentially slow the HIV epidemic. However, despite its promise, PrEP uptake remains suboptimal with a limited scale of application in regions of the world that would benefit most from its use [7].

By 2021, approximately one million individuals in SSA have received PrEP, most of whom were enrolled as part of clinical trials and demonstration studies [1, 7]. Current knowledge around the context of PrEP uptake has been largely shaped by these studies, which are often conducted under ideal conditions and among priority groups, such as sex workers and Men who have Sex with Men (MSM) [8]. As countries move towards the application of PrEP in the general population, insights regarding individual-level motivation for PrEP use, and rationales for PrEP decline are essential to (1) inform the design of targeted PrEP programs that match user priorities and generate demand; and (2) modify existing PrEP programs to better suit the needs of the target audience.

Lesotho has a generalized HIV epidemic and an adult HIV prevalence that is among the highest globally [9]. To address this high disease burden and curb new cases, the Ministry of Health incorporated PrEP as a prevention strategy into the national HIV program in 2016 [10]. When first introduced, the program prioritized demand creation and enrollment among serodiscordant couples. It later broadened its scope to include critical populations and, as of 2020, PrEP was offered to all persons categorized as being at high risk for HIV infection. In a phased implementation approach, the Lesotho PrEP program was first rolled out in five of the country’s 10 districts [10]. Currently, only an estimated 15,749 Basotho people have been enrolled on PrEP [9]. This suggests a need for investigation into low PrEP uptake and investment in informed demand creation activities based on these findings.

This present study sought to understand the context of PrEP uptake and decline in a general population experiencing hyperendemic levels of HIV in Lesotho. This work aimed to inform local program implementation and offer insights to other programs in similar settings globally. The study aimed to: (1) describe PrEP user demographic profiles; (2) understand motivations behind PrEP use; (3) report PrEP enrollment experiences; and (4) detail reasons for PrEP decline among those identified as being at risk for HIV infection.

## Methods

### Study design and respondent sampling

This study was embedded within a larger implementation project conducted in collaboration with the Lesotho Ministry of Health. The overarching study aimed to examine the initial implementation phase of the PrEP program to inform national scale-up [11]. This present study, conducted between March and May 2019, was informed by Grounded theory [12] and employed qualitative methods (Table 1). In-Depth Interviews (IDIs) were conducted with purposively selected key decision makers at the policy level directly involved in designing and implementing the national PrEP program. They included Ministry of Health officials at the national and district levels and officials from Non-Governmental Organizations (NGOs) tasked with implementing the program. To these policy-level respondents, we posed questions on their overarching views on PrEP demand, trends in uptake and reported reasons for the drug’s decline.

**Table 1:** Data collection activities conducted as part of this present study

Data collection activity	Respondent groups	Number
Semi-structured In-Depth Interviews	Policymakers and implementing partners	9
	Current users	55
	Former users	36
	Decliners	6
Focus group discussions (FGDs)	Health providers	11*
Total qualitative data collection activities		117

^*^With a total105 participants

Interviews were also conducted among individuals with direct experience using or being offered PrEP and falling into one of three categories: current, former, or decliners. Current users were defined as respondents who were using PrEP at the time of the interview, regardless of the duration of use. Former users, on the other hand, were defined as individuals who, at one time, had used PrEP but were not using the drug at the time of the interview. The duration of nonuse was not used as an inclusion/exclusion criterion for participation in the study. Decliners were defined as individuals who were offered PrEP by a health provider owing to their high risk of HIV acquisition, yet they refused to use PrEP. End-users were all identified through health facility records and community-based organizations working with key populations. Interview questions with end-users were designed to capture personal experiences of enrolling in or declining use of PrEP.

Focus Group Discussions (FGDs) with health providers were conducted to gain their perspectives on PrEP demand, uptake, and decline, based on their experience with clients. Providers were selected to participate in FGDs based on their direct provision of HIV and PrEP services. Given their professional standing, gender was not viewed as influencing full participation of health providers in FGD. Therefore, both male and female health providers were included in the same FGD.

### Study sites

**Fig. 1:**
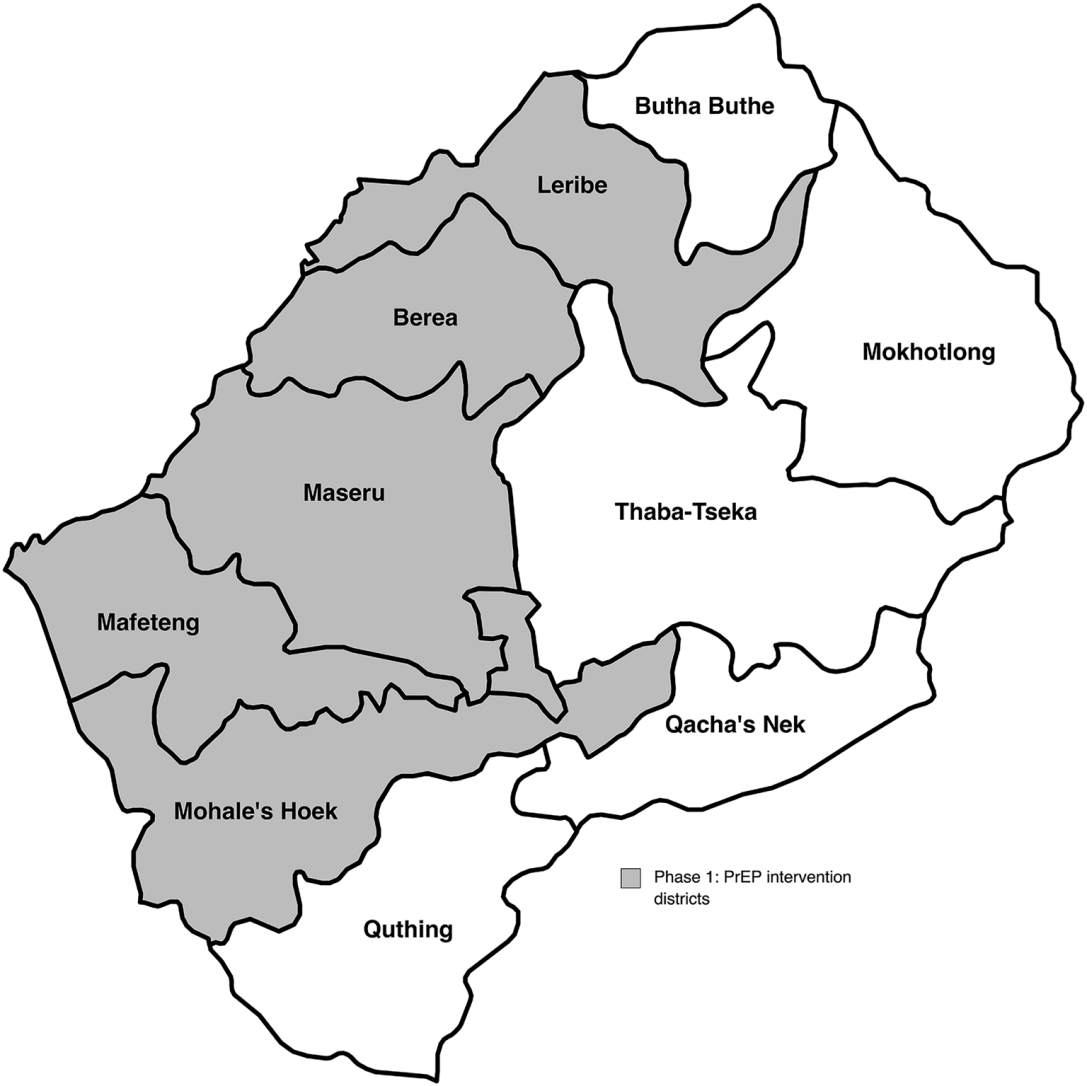
Lesotho’s national PrEP implementation districts: Phase 1

Sites included in this study were selected from the five districts that were part of the first phase of PrEP implementation: Maseru, Leribe, Berea, Mafeteng and Mohales Hoek (Fig. 1). To maximize sample size, national PrEP enrollment and distribution records were reviewed, and high-volume facilities in each district were prioritized for inclusion in the study.

### Data collectors and training

Experienced Research Assistants (RAs) with undergraduate and graduate training in the social sciences were recruited to conduct IDIs and FGDs. Prior to the start of data collection, RAs undertook a three-day training, which reviewed the aims of the study, qualitative research methods, and research ethics. Pilot testing of the study instruments was also included outside the selected study sites.

### Data management and analysis

Interviews and discussions were digitally audio recorded with the respondents’ permission. The resulting recordings were transcribed and selectively translated into English by the same interviewers, where possible.

Data analysis was initiated in the field, with debriefing meetings held every day following each interview/FGD and led by the first author [13]. During debriefings, an investigator systematically led RAs to review the collected data, identify new areas for deeper exploration, and discuss topics that have achieved saturation. Following the end of data collection, a formal analysis workshop was held to elicit feedback from RAs. During this workshop and in discussions with investigators, major themes emerging from the data were identified. Codes were then developed to reflect these themes and collated into a codebook. These codes were applied to all transcripts using Atlas.ti [14]. Data under each code was summarized, and quotes were selected to illustrate emerging themes.

## Results

### Study population

Our study included 106 in-depth interviews with policymakers (n = 5), PEPFAR implementing partners (n = 4), and PrEP end-users (current users = 55, former users = 36, decliners = 6). Duration of PrEP use among current end-users ranged between two days and 31 months, with an average use of eight months. Former PrEP users had been on PrEP for an average of 4 months (range three days–24 months) prior to discontinuation. Eleven FGDs involving 105 health providers offering HIV and PrEP services were conducted.

### PrEP demand

Individuals at imminent risk of HIV infection were identified as being most interested in initiating PrEP, with couples tested together and found to be serodiscordant described as more likely to seriously consider initiating the drug regimen. Health providers and implementing partners identified adolescents and young adults as being the age group most interested in PrEP. However, providers described the youth as presenting ethical challenges because of assent/consent, particularly in instances where the adolescents were under the care of their parents. On a personal level, health providers and parents alike struggled with whether PrEP promotion in this age group sends the *“wrong message”* and assumes that children are engaged in sexual relationships. One provider recounted:

“*In most cases parents refuse when their children are offered PrEP because they consider their children as young and not ready to be engaged in sexual activities. It is not easy to engage in sex talk in our culture and the parents still do not believe their children can engage in sexual activities and yet those children have already started.”*—Health provider FGD, urban setting, location 3

### Enrollment experiences and counseling

Enrollment procedures were overwhelmingly described as important and in a positive light by both end-users and health providers (Table 2). For health providers, the enrollment period, including initial counseling, was described as a time to create rapport, assess PrEP eligibility including risk for HIV infection, discuss what PrEP use entails if the client is eligible, and devise alternative HIV prevention avenues should the client decline PrEP. During the initial counseling session, both health providers and clients described PrEP’s ability to prevent HIV infection as being the central point of discussion. Moreover, clients reported PrEP as being marketed to them as a tool to protect themselves and their loved ones from the burden of HIV, and something each client must choose for themselves. As one health provider explains:

“*The first and most important thing, is to give a client health education, explaining everything about PrEP, what its benefits are. And then we address the client-specific situation, giving them the chance to make a fully informed decision on whether to start using PrEP.”*—Health provider FGD, rural setting, location 10

**Table 2:** Summary of reported purpose and experience of initial counseling and enrollment

Health providers	End-users	
	Positive experiences	Negative experiences
• Assess PrEP eligibility; • Build rapport, establish trust, and form relationships with clients; • Explain PrEP purpose and modalities of use; • Address and allay concerns from the client; • Give client opportunity to enroll on PrEP if eligible; • Triage ineligible clients to other services at health facility; • Discuss alternative HIV prevention avenues with decliners	• Gain greater understanding of PrEP; • Avenue to ask questions; • Emotionally process partner’s HIV positive status; • Deliberate usefulness of PrEP for their personal situation	• Little to no counseling, leading clients to believe they were HIV positive; • Pressure to enroll on PrEP

One-on-one counseling was viewed as a safe space to process emotions and difficult news for couples who discovered one partner is HIV positive. Ongoing counseling was described as building client-provider trust and relations, should additional queries arise. For end-users, pre-enrollment counselling was seen as an opportunity to gain greater understanding and information about PrEP, ask questions for clarification, allay misconceptions and deliberate if PrEP will be useful for them and their situation. As one current user explained:

“*It is because my health provider was patient with me. She started explaining from the little knowledge I had with PrEP and took me step by step because I didn’t know anything about it. That is what made counselling work for me.”*—Current PrEP user, 63 years old, male, urban setting

In some cases, respondents reported insufficient or superficial counseling that did not meet the client’s expectations, leading end-users to be misinformed about their diagnosis. Four current PrEP users we encountered had assumed they were HIV positive after receiving pills following testing with their partners. These respondents only came to learn about their true diagnoses at follow-up meetings at the health facility or following our interview. This poor counseling was attributed to health providers having insufficient time to discuss PrEP in detail with the client during disbursement. When counseling was not satisfactory, some respondents decided to go to another health facility, go back to the health facility when the “*problematic*” provider was not there, or move from community providers to a health facility to get more in-depth counseling.

Differences in the quality of counseling provided at the community and facility levels were discussed by policymakers and health providers, albeit tactfully. Respondents were careful not to disparage the community PrEP partners while highlighting areas of improvement in message delivery. One health provider explained:

*“Here at the facility, we have various people who pass on knowledge, from the counsellor to the clinician to the pharmacist, all of whom work to ensure that the client is well informed before taking on the drugs. That is very different from the community, where it seems the health educators are just pushing their own agenda. It seems like clients are just pushed on PrEP. I’m not judging, but it seems that clients are rarely properly informed.”-*—Health provider FGD, urban setting, location 2

One decliner echoed this push for enrollment numbers by reporting feeling “*pressured*” to take home PrEP pills, despite feeling unprepared or ineligible for the drug. The respondent decided to *“just take the pills and flush them in the toilet*”. She received a month’s dose of PrEP and kept them in her house, without using any. When providers followed up with the respondent, she initially made excuses to say she was busy, and subsequently ignored their phone calls. Policymakers and implementing partners echoed this experience, explaining they have received reports of clients being provided with PrEP yet not using the drugs.

### Motivations for PrEP use

#### Serodiscordant relationships

Approximately half of our end-users reported PrEP initiation owing to a sexual relationship with a seropositive partner (Table 3). Health providers and implementing partners reaffirmed the use and demand in this population, noting they recommend PrEP use especially when a sexual partner has recently been diagnosed as HIV positive, and/or is in the acute phase of infection, and/or has not achieved viral suppression. For the negative partner, PrEP was described as providing a sense of security and normalcy in their sexual relationship, with concern of HIV infection being greatly diminished. This was emphasized in respondents who were in committed relationships and were unwilling to dissolve their relationship because of an HIV diagnosis.

“*My husband was using ARVs for some time, which was making it difficult for us to have sex as often as we wanted to, because there was nothing that was defending me from getting HIV. But when I heard of PrEP, I was very happy because I felt I had found my solution.*”—Current PrEP user, 36 years old, female, rural setting

**Table 3:**
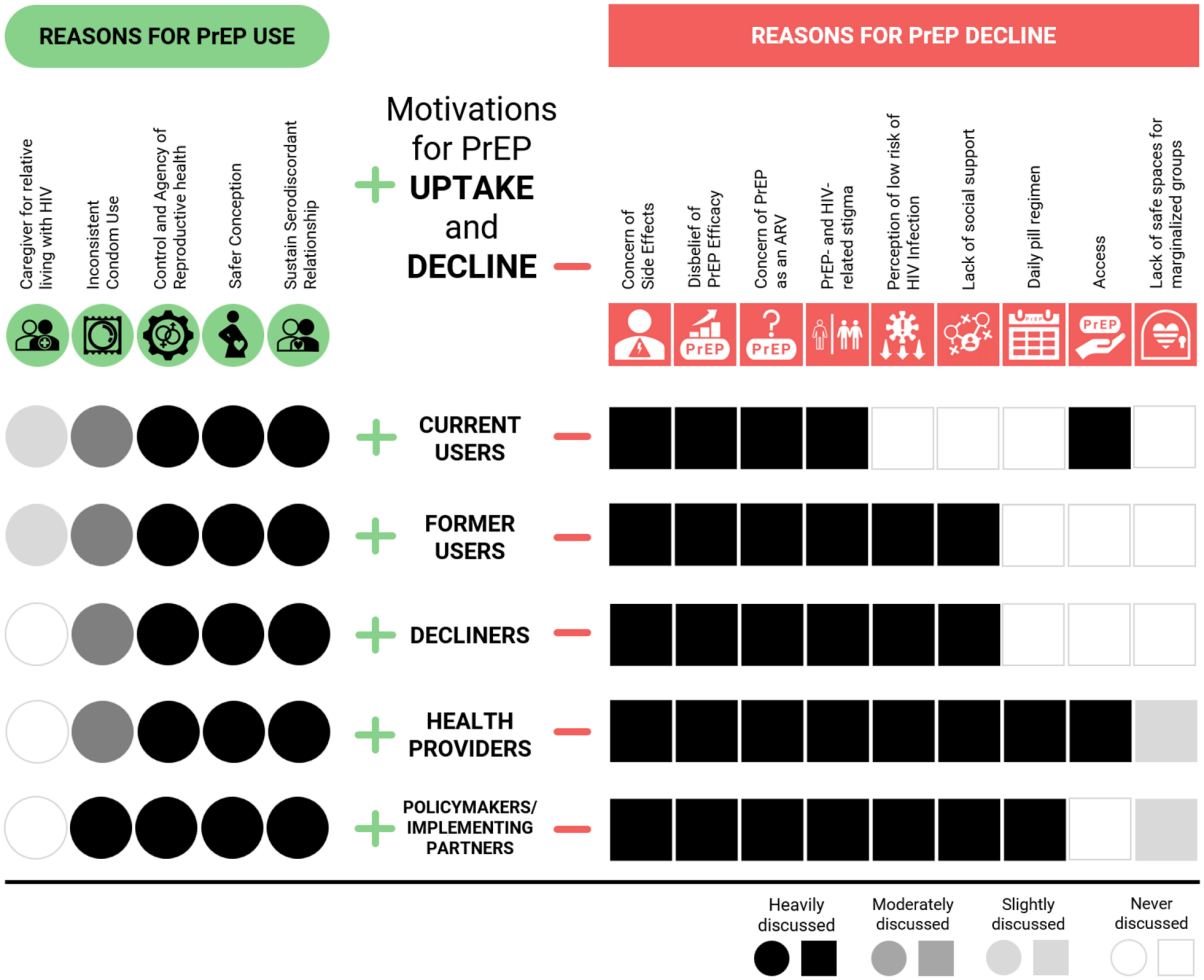
Motivations for PrEP uptake and decline provided by study respondents

Furthermore, initiation on PrEP offered comfort to serodiscordant couples with children, who intended to ensure they remain healthy for the sake of their offspring. Urgency of PrEP use was further exacerbated in the case where the seropositive partner was reluctant to begin treatment. Several respondents described scenarios where their partners were in denial of their HIV status, consequently refusing to begin Antiretroviral Therapy (ART). They therefore used PrEP as they awaited their partner to come to terms with their status. One current user explained:

*“My husband doesn’t want to take ARVs. I have been encouraging him, but he has not listened to me. I thought that I will start taking PrEP so that I will be able to look after our children.”*—Current PrEP user, 33 years old, female, rural setting

#### PrEP as a means for safer conception

For couples and individuals whose intention is to grow their family, PrEP was viewed as providing and/or restoring hope for having children. The prophylactic provided for a longer window for conception, and diminished fear of infection while trying to conceive. As one current PrEP user explained:

“*I no longer use ‘protection’ since I started using PrEP. I stopped using [condoms] because we were planning to have a second born.”*—Current PrEP user, 33 years old, female, urban setting

#### PrEP use as an avenue for control of sexual health

Lack of trust in the fidelity of sexual partners emerged as a recurring theme for PrEP use across all respondent groups. End-users reported suspicion and/or discovery of their partner engaging in multiple and concurrent relationships as an impetus for initiating PrEP. Behavior signaling infidelity included a partner arriving home late, evidence of extramarital affairs (including children conceived through those relationships), migrant work leading to extended periods of time away from home, and partners who did not voluntarily avail themselves for HIV testing. One respondent who is currently using PrEP explained:

“*I thought that since I do not know what is happening with my partner while I am away, I decided to visit the facility and ask more about PrEP. I like testing every so often just to be sure of my status. But my boyfriend refuses to test whenever I ask him to, and I know that he has some loose behavior.*”—Current PrEP user, 30 years old, female, urban setting

PrEP was also discussed as a means of protection where there was uncertainty of a partner’s HIV status, particularly in newly formed relationships where there was trepidation in addressing this subject directly. A health provider noted:

“*My client was a widow, and she suspected that her new ‘friend’ was HIV-infected. But she didn’t dare ask him directly. She was not sure on how to protect herself, but when she learned of PrEP, she was very quick to get initiated.*”—Health provider FGD, urban setting, location 4

Secret PrEP use among individuals in the sex industry– that is use of PrEP by sex workers without the knowledge of their clients—was reported among those who either had little agency in negotiating for condom use during transactional encounters, were concerned about condom failures (like breakage, or removal during intercourse), or were asked to engage in condomless sex at a premium. A former PrEP user explained:

“*I am exposed to so many risks because I meet so many men in my work. So it might happen that sometimes a condom gets torn. Or sometimes I get to meet that person who will give me extra money if I do not use a condom.”*—Former PrEP user, 49 years old, female, urban setting

#### PrEP to circumvent inconsistent condom use

The inability or dislike of condoms use was reported by all respondent groups as a key reason for PrEP initiation. Health providers noted that although condoms have been available and promoted for safer sex in Lesotho for decades, their personal experience with clients has shown a lack of enthusiasm for this barrier method. For providers, suboptimal condom use is evident in the stagnating HIV incidence rates in the country. Across all respondent groups, PrEP was discussed as an added layer of protection when condoms were not an option, and more so as a realistic option for protection that can be used more consistently. A current PrEP user explains, “I do not use a condom in order to satisfy [my partner]”. She therefore uses PrEP to protect herself instead. A health provider elaborated further:

*“We know that men dislike using condoms, but like having sex. But for those women who know that their partners dislike using condoms, then PrEP is a very good option. With PrEP, at least both parties will be able to protect themselves.”*—Health provider FGD, urban setting, location 4

Some respondents described PrEP use as a harm reduction solution, acknowledging that although the drug does not protect against STIs, it protects against HIV, which has deadlier perceived outcomes. Therefore, forgoing condom use whilst using PrEP was viewed as the lesser of two evils. However, both end users and health providers noted the quandary some individuals find themselves in when deciding to disclose their PrEP use. In relationships lacking open communication, users reported keeping their PrEP use to themselves, cautious that their partners might abandon condom use all together. As one current PrEP user explains her situation, “After telling [my partner] that I am using PrEP, he refuses to use condoms now.”

#### Caregiver for a relative living with HIV

In a minority of cases, end users reported the use of PrEP due to concern of exposure from caring for HIV positive family members. One former PrEP user said:

*“[I decided to start PrEP] when I was taking care of my sick sister. She was HIV positive, and she had open wounds. I couldn’t bathe her with gloves on because it was like I was hurting her. I sometimes had to bath her without gloves and that put me at risk.”*—Former PrEP user, 23 years old, female, urban setting

### Reasons for decline of PrEP initiation/enrollment

Several reasons were discussed as contributing to the decision to refuse PrEP initiation among the six decliners and other respondents enrolled in our study (Table 3). Of note, PrEP decline was seldom because of a singular reason. Rather, a confluence of factors and/or personal circumstances led respondents to the decision to decline enrollment on PrEP.

#### Side effects

Half of our respondents who declined PrEP (n = 3) did so due to concern about the drug’s side effects—including nausea, headaches, dizziness—which they have heard from friends using it. As one decliner explained:

*“I was with a friend of mine having a drink. She told me that she has been using PrEP and that it caused her to have dizziness and to vomit… I did not take PrEP because of the side effects my friend went through.”*—PrEP decliner, 30 years old, female, urban setting

#### Drug efficacy

In addition to disconcerting adverse effects of PrEP, two decliners in our sample cited being unsure about the extent to which the pill works to protect against HIV infection as one of the reasons they personally declined taking up the prophylactic drug. The question of PrEP’s efficacy was also highlighted as a reason for PrEP decline up by all other respondent groups. A respondent who first heard of PrEP during a community event recounted her experience and rationale for decline, stated:

“*…I don’t believe in these things that are said to prevent HIV. I thought that this pill is the one that is increasing the chances of getting [HIV] infection. I don’t like PrEP. I don’t even want to know more about it … Like I said, I don’t believe in these pills that are said to decrease chances of people getting infected*.”—PrEP decliner, 23 years old, female, urban setting

#### Concern of ARV use for HIV negative individuals

Both decliners in our sample, as well as other respondents reported concern of PrEP use because the drug is composed of ARV pharmaceutical ingredients. Respondents lamented that they could not understand how a drug used for HIV treatment can also be used for its prevention:

*“Well, I thought [PrEP] will help me when I first heard about it. But then I decided not to take it because I heard it was an ARV… I would love to know how it prevents HIV, but it is another part of ARV.”*—PrEP decliner, 35 years old, female, urban setting

Concern over PrEP’s pharmaceutical composition was of particular concern for breastfeeding women, who feared that the ARVs would affect their nursing child. A health provider recounted her experience with such a client:

*“I have encountered one client who upon finding out that PrEP contains ARVs, she promptly refused to enroll on it. She was very shocked to learn that they are still ARVs, and she was a breastfeeding mother. She had initially thought it was just a drug. It sounded like she knew what PrEP is and what it does but was not aware of its chemical composition. And that’s what turned her off.”*—Health provider FGD, urban setting, location 8

#### Stigma

While concern that PrEP is an ARV was a partial reason for its decline, the prospect of others conflating PrEP use with a positive HIV status was an additional point of concern for prospective clients. For respondents in the sex industry, this conflation posed substantive financial consequences:

*“…I am a sex worker. This is how I earn a living… if I use PrEP, I am afraid I am going to lose my customers because they are going to say I am using ARVs.”*—PrEP decliner, 32 years old, female, urban setting

The issue of stigma was further compounded by the fact that PrEP users are required to line up and collect their pills from the same area as those collecting their ARVs, and the fact that PrEP and ART drugs resemble in appearance and packaging. An implementing partner noted:

“*It has been a little difficult to promote PrEP because it is an ARV. People associate it with being HIV positive. When someone goes to collect the pills, they are afraid they will be labeled as being HIV positive.*”—Implementing partner #2

This concern was corroborated by a health provider who noted that people were afraid of being labeled “*ba phaka*”, meaning one who is taking ARVs. During a discussion, one health provider recalled a client who had agreed to enroll on PrEP. As they were in line to pick up the drugs, the client noticed that they were in line with people waiting for ART and decided to leave. They did not want to be on the same queue as people picking up ART.

#### Perceived risk

One decliner noted that she did not view herself as being at risk of HIV acquisition, because she was not involved in concurrent relationships. She explained:

*“There were people from [an NGO] who came to our village. We got tested [for HIV] and they offered us PrEP. But I declined because we were told it is taken by people who are in multiple relationships. Since I have one partner, I did not see any use of taking it at all.”*—PrEP decliner, 22 years old, female, urban setting

#### Lack of familial and social support

Decliners noted that lack of support, and more so, discouragement by a close family member or friend results in decline. One decliner narrated her experience:

*“My husband discouraged me to take [PrEP]. He shouted at me as I was taking it and said that [PrEP] will give me an HIV infection… I vomited it out immediately I took it. I had faith in [PrEP] but my husband discouraged me.”*—PrEP decliner, 35 years old, female, rural setting

The importance of support from one’s social network was reaffirmed by other respondent groups. A current user noted:

*“The problem comes when you talk to someone who will discourage you from taking PrEP, people will tell you that there is no need for you to be taking PrEP when you don’t have HIV.”*—Current PrEP user, 63 years old, male, urban setting

#### Access to PrEP

Challenges associated with accessing PrEP—including distance from health facilities and transportation costs—were discussed as contributing to the decline of PrEP in clients who would benefit from using the drug. Health providers in particular recounted encounters with prospective PrEP users who expressed access as a significant barrier. However, some providers described this barrier as being a superficial one. In a more nuanced explanation, health providers in one FGD noted a lack of urgency and the perception of minimal HIV infection risk as underpinning access barriers. This health provider explained:

*“Others say they do not want to start PrEP because they live and work far away from [a health facility], which makes it difficult for them to come back here to attend their appointments every month. Even with those busy working here in [this town], if their drugs run out, they don’t feel compelled to go to the nearest facility to pick up their PrEP drugs. Because for them, they are still HIV-negative, so there is no real desire. This is unlike ART clients who know that if they stop their medication for a certain period, their viral load could go high.”*—Health provider FGD, rural setting, location 6

#### Pill regimen

The prospect of taking a pill every single day, compounded by the fact that an individual may be on PrEP for a long duration, was reported as a deterrent to initiation. Health providers discussed having had several clients who after undergoing counseling opted to decline PrEP initiation for this reason. One health provider noted that men do not like the idea of taking a pill every single day. A district level respondent noted she received a report of a sex worker who after hearing she would need to be on PrEP for 3 months at minimum, she responded saying she was not sick and there was no need to take drugs for such a long time. One implementing partner explained further:

“*The perception is that PrEP is like antibiotics—you take it for 7 days or 21 days; or even like TB medication, which you take for 6 months. When patients hear, you might have to take it for the entire time you are at risk for HIV, which might be a lifetime, they decline. Clients wonder, “I am not sick, why take [PrEP]? [And] I still have to use condoms? What’s the use?*”—Implementing partner #3

#### Lack of safe spaces for marginalized/underrepresented groups

Providers noted that the MSM community in their catchment area were interested in PrEP. However, they did not like coming to health facilities because there were not safe spaces for them. They reported feeling discriminated against at male clinics, and they do not feel comfortable in other areas that are considered female spaces. The providers explained that these concerns led MSM to look for a supply of PrEP outside the clinic on the black market where 1 pill can cost up to 200 rand (12USD). A health provider noted:

*“As much as we are a Christian country propagating Christian values, we also need to realize that LGBTI groups exist among us. They live among us, some of them are even our husbands. In the same way that we have male clinics, we also need to open an LGBTI friendly clinic just for them, so that we provide user-friendly services at user-friendly spaces.”*—Health provider FGD, rural setting, location 5

## Discussion

In this qualitative implementation study, we aimed to contextualize and draw useful lessons from PrEP uptake in Lesotho. Our findings indicate that initial counseling and engagement prior to PrEP enrollment is crucial for both health providers and end-users. This time was discussed as useful for not only information transfer, but also as a means of building trust and addressing user concern. Poor counseling and/or pressure to enroll was seen as generating distrust and disinterest in PrEP. Motivations for PrEP uptake centered on the strengthening of the family unit—through bolstering serodiscordant relationships, providing for safer conception or caring for ailing relatives—and promoting agency over personal sexual health—brought about by infidelity, uncertainty of partner HIV status and dislike of, and/or inconsistent condom use. PrEP decline was driven by individual-level factors (risk perception, perceived side effects, disbelief of the drug’s efficacy and daily pill regimen), societal factors (conflation of PrEP use and HIV positive status, lack of familial/social support, lack of safe spaces for marginalized groups and HIV-stigma), and structural factors related to PrEP access.

Youth and women were described by our respondents as being most interested in enrolling on PrEP. However, this interest did not necessarily translate to enrollment, as demonstrated in our work and others [15]. Given that adolescent girls and young women in sub-Saharan Africa are disproportionally affected by HIV [16], there is an opportunity for greater demand creation and targeted messaging to encourage enrollment in this population. Growing evidence in the field promotes deviation from risk-based messaging for PrEP demand creation, with recommendations for movement towards more positive framing [8, 17]. This move is supported by documented challenges of assessing the risk of HIV infection. While our respondents discussed PrEP demand in relation to perceived risk, literature suggests individuals gravely underestimate or rationalize their risk of HIV acquisition [18–20]. Because risk is difficult to self-assess, it suggests that a PrEP demand campaign that focuses on the motivators of PrEP use identified here and in other literature would be more beneficial. Our findings and other studies have shown similar stimuli for PrEP use—family, safer conception and agency [21–23]. These aspects of PrEP could be leveraged in positive messaging and demand creation. Further still, in the messaging of PrEP to young women of reproductive age, tailored messaging is required to allay concerns over harm to breastfeeding a child and/or fertility [24].

During the initiation phase, comprehensive and deliberate counseling emerged as key to equipping the end user with the knowledge necessary for decision making for PrEP uptake. In addition, this time offers a reasonable period for dynamic dialogue where the health provider could respond to user needs. Specifically, these sessions can be used to address—at the individual level—misinformation and/or concerns held by the prospective user. Of note, our work and others show distrust of PrEP’s efficacy as a substantial barrier to PrEP uptake [25]. More generally, the idea that one pill can prevent HIV has been shown as a difficult concept to reconcile [25].

Findings from our work show that structural barriers related to access contribute against PrEP initiation, like in other parts of the continent [26]. However, our respondents noted that these structural factors might not act in isolation, and may be surmountable, if the user is keen on PrEP use. These structural factors may be a symptom of other more pressing barriers. Our findings further underscore the importance of considering the social context while introducing PrEP. Most poignantly, gender dynamics and HIV-related stigma emerged as social barriers of paramount importance. Understanding and accommodating complexities related to decision making may improve PrEP uptake. Men have been found to discourage their partners from initiating and adhering to PrEP [27–29]. We can learn from HIV treatment programs and PrEP demonstration studies, which have identified the importance of engaging men in HIV-related programing [27]. Male allyship is critical beyond PrEP uptake and retention—cooperation of men is required to ensure continued condom use in addition to PrEP use. Our study and others have demonstrated the dilemma resulting from PrEP promotion that can lead to risk compensation—increased risk taking, including condom non-use and increased sexual partners, due to perceived protection from PrEP [30]. Therefore, tactful, and informed PrEP initiation and continued counseling is required to dissuade risk compensation.

Perceived and/or anticipated stigma was discussed as a barrier for PrEP uptake here and in other studies [31–34]. This may have resulted from messaging around PrEP as a drug for use by those with multiple partners, sex workers and MSM in sub-Saharan Africa. This framing draws attention to, and questions the “morality” of the user. Stigma was also described as resulting from conflation of an individual on PrEP as being HIV-positive owing to accessing PrEP services at the same location as those receiving HIV treatment services, and the similarity in appearance of PrEP and ART [35]. Given this context, a concerted effort to distinguish PrEP and ART is key. This could include discrete packaging, which is visibly different from ART, and physically separating PrEP and ART delivery service points.

### Strengths and limitations

This study was limited by the low number of decliners (n = 6) who participated in the study. Despite intensive efforts to identify and recruit more decliners, we were unable to do so, because information on decliners is not recorded and therefore not readily available. Future studies would benefit from recruiting more participants in this population to more accurately capture the experience of those who decline PrEP use. Additionally, we relied on our participants’ memory for descriptions of the enrollment process, counseling and general PrEP experiences. This may have introduced some recall bias. Future studies may benefit from triangulation of IDIs and FGDs with other research methods, such as observations and exit interviews. The strength of this study is its inclusion of diverse stakeholders having vested interest in the national PrEP program. Our work reflects the complexity of program implementation from multiple vantage points, with particular attention to end-user experiences. This work has immediate implications and applicability to the Lesotho PrEP program, and other similar programs on the African continent and beyond.

#### Study implications

Our findings provide important recommendations and implementation strategies for rolling out PrEP in HIV-hyperendemic settings. First, targeted PrEP messaging and demand creation activities is required prior to the rollout of a national PrEP program, and throughout the program’s implementation. Our study suggests that a campaign highlighting PrEP’s potential of enhancing personal sexual health agency and strengthening familial bonds may encourage its uptake. Additionally, messaging directly addressing the individual-, social- and structural-level motivations for PrEP decline may also increase the desire for PrEP initiation. Second, health worker training on comprehensive counseling as part of the PrEP initiation and enrollment process is essential. Our work showed that PrEP initiation seldom occurs in a one-off meeting, but rather is an ongoing relationship between the health provider and client where the client makes the decision to begin PrEP use over time. Lastly, on the health system and global level, distinction of HIV prevention and treatment programming is needed to minimize HIV-related stigma and encourage PrEP initiation. Participants in our study suggested differentiated packaging and appearance of PrEP and ART drugs would achieve this distinction. Additionally, removal of HIV-only sections in health facilities would likely lessen stigma associated with seeking HIV prevention and treatment services.

## Data Availability

Not applicable.
